# Human SARS-CoV-2 has evolved to reduce CG dinucleotide in its open reading frames

**DOI:** 10.1038/s41598-020-69342-y

**Published:** 2020-07-23

**Authors:** Yong Wang, Jun-Ming Mao, Guang-Dong Wang, Zhi-Peng Luo, Liu Yang, Qin Yao, Ke-Ping Chen

**Affiliations:** 1 School of Food and Biological Engineering, Jiangsu University, 301 Xuefu Road, Zhenjiang, 212013 China; 2 Institute of Life Sciences, Jiangsu University, 301 Xuefu Road, Zhenjiang, 212013 China

**Keywords:** Evolutionary genetics, Influenza virus

## Abstract

The outbreak of COVID-19 has brought great threat to human health. Its causative agent is a severe acute respiratory syndrome-related coronavirus which has been officially named SARS-CoV-2. Here we report the discovery of extremely low CG abundance in its open reading frames. We found that CG reduction in SARS-CoV-2 is achieved mainly through mutating C/G into A/T, and CG is the best target for mutation. Meanwhile, 5′-untranslated region of SARS-CoV-2 has high CG content and is capable of forming an internal ribosome entry site (IRES) to recruit host ribosome for translating its RNA. These features allow SARS-CoV-2 to reproduce efficiently in host cells, because less energy is consumed in disrupting the stem-loops formed by its genomic RNA. Notably, genomes of cellular organisms also have very low CG abundance, suggesting that mutating C/G into A/T occurs universally in all life forms. Moreover, CG is the dinucleotide related to CpG island, mutational hotspot and single nucleotide polymorphism in cellular organisms. The relationship between these features is worthy of further investigations.

## Introduction

The outbreak of COVID-19 (coronavirus disease 2019) was listed as a public health emergency of international concern on 30 January 2020 and declared a global pandemic on 11 March 2020 by the World Health Organization. As of 15 June 2020, it has caused more than 7.8 million infection cases and over 430,000 deaths worldwide^1^. Its causative virus (officially named SARS-CoV-2) has a genome of single-strand positive-sense RNA with approximately 30,000 nucleotides^2^. Based on its genome sequence, analyses have been conducted to characterize genomic features and to trace origin of the virus^3,4^. Meanwhile, many reports have been focused on developing efficient methods for detection^5,6^ and screening effective drugs for treatment^7,8^ against COVID-19.

Coronaviruses have some of the largest RNA genomes among all viruses. The single-strand genomic RNA of coronavirus has a cap-like structure at 5′-UTR (untranslated region) and a poly(A)-tail at 3′-UTR, both of which allow it to assume a structure similar to mRNA of host cells. After being released into cytoplasm of a host cell, the viral RNA is translated immediately to produce viral proteins by using the translation machinery of host cells^9^. The viral RNA is much longer than host mRNAs. Such a long single-strand RNA will form multiple stem-loops through base-pairing between its adjacent segments. These stem-loops present an obstacle to the translation machinery, because they must be disrupted to expose coding information contained in them. The stability of a stem-loop structure is dependent on number of hydrogen bonds formed between bases in the stem part. Because C-G and T-A base-pairs are formed through three and two hydrogen bonds respectively, a viral RNA strand with high number of C and G bases will form more stable stem-loops than that with high number of T and A bases.

In order to understand whether translation of coronavirus RNA is greatly affected by base composition, we analysed dinucleotide distribution and RNA stability of twenty-four coronavirus species. We found that open reading frames (ORFs) of SARS-CoV-2 have an extremely low abundance of CG dinucleotide. Moreover, the secondary structure formed by SARS-CoV-2 genomic RNA is less stable than many other coronaviruses. Therefore, it is suggested that SARS-CoV-2 is more efficient in reproduction than other coronaviruses, because less energy is consumed in disrupting the stem-loops formed by its genomic RNA.

## Results

### Low CG content in human SARS-CoV-2

**Figure 1:**
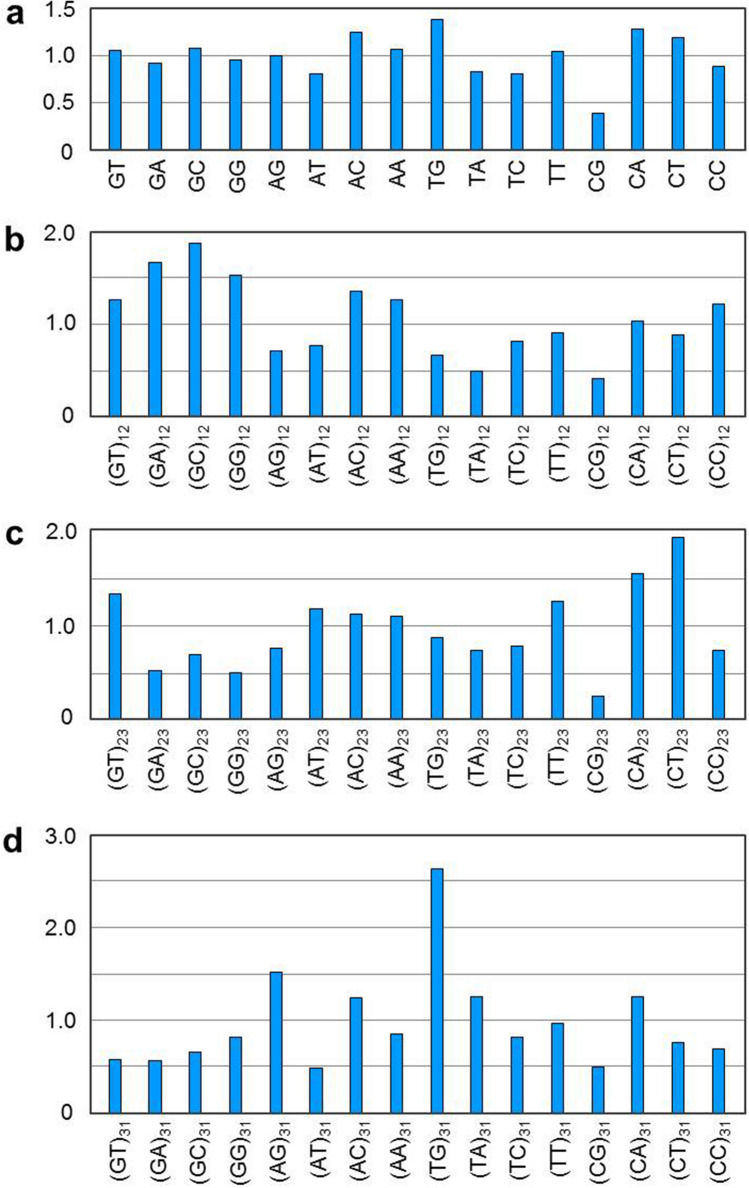
Odds ratios of dinucleotides in open reading frames of SARS-CoV-2. (**a**) odds ratios of dinucleotides at all codon positions. (**b**–**d**) odds ratios of dinucleotides at codon positions 1 and 2, 2 and 3, 3 and 1, respectively. Value shown in the figure is weighted average odds ratio of each dinucleotide. Odds ratio of each dinucleotide in ten ORFs (i.e. ORF1ab and ORF 2–10) of SARS-CoV-2 is calculated respectively first. Then, a weighted average odds ratio is obtained based on length of each ORF.

DNA or RNA sequences are composed of four nucleotides, i.e. adenylate (A), thymidylate (T), guanylate (G) and cytidylate (C). They can also be considered polymers of 16 dinucleotides. Odds ratio is a value defined to indicate relative abundance of a nucleotide, which is the ratio of observed to expected frequency of a dinucleotide^10^. The genome of SARS-CoV-2 (29,903 nucleotides^2^, sequence number NC_045512) has 29.94% of A, 32.08% of T (T is used here instead of U for simplicity), 19.61% of G and 18.37% of C. Thus, the expected frequency of CG dinucleotide in viral genome is 3.60% (i.e. 19.61% × 18.37%). However, only 439 CGs are observed, which means the observed frequency of CG dinucleotide is 1.47% (i.e. 439/29,902). Therefore, odds ratio of CG in SARS-CoV-2 is 0.41 (i.e. 1.47%/3.60%). Furthermore, odds ratio of CG in open reading frames (ORFs) of the virus is 0.39, being the lowest among 24 coronaviruses under survey (Fig. 1a and Table S1). Because a codon is composed of three nucleotides, a dinucleotide (e.g. CG) has three possible locations. Herewith, they are designated as (CG)_12_, (CG)_23_ and (CG)_31_ respectively. We found that the odds ratio of (CG)_23_ in ORFs of SARS-CoV-2 is as low as 0.25, while that of (CA)_23_ and (CT)_23_ is as high as 1.54 and 1.92 respectively (Fig. 1c). Moreover, odds ratio of (CG)_31_ in ORFs of SARS-CoV-2 is 0.50, while that of (AG)_31_ and (TG)_31_ is 1.52 and 2.64 respectively (Fig. 1d). These data strongly suggest that (CG)_23_ has been mutated into (CA)_23_ and (CT)_23_, and (CG)_31_ has been mutated into (AG)_31_ and (TG)_31_.

**Figure 2:**
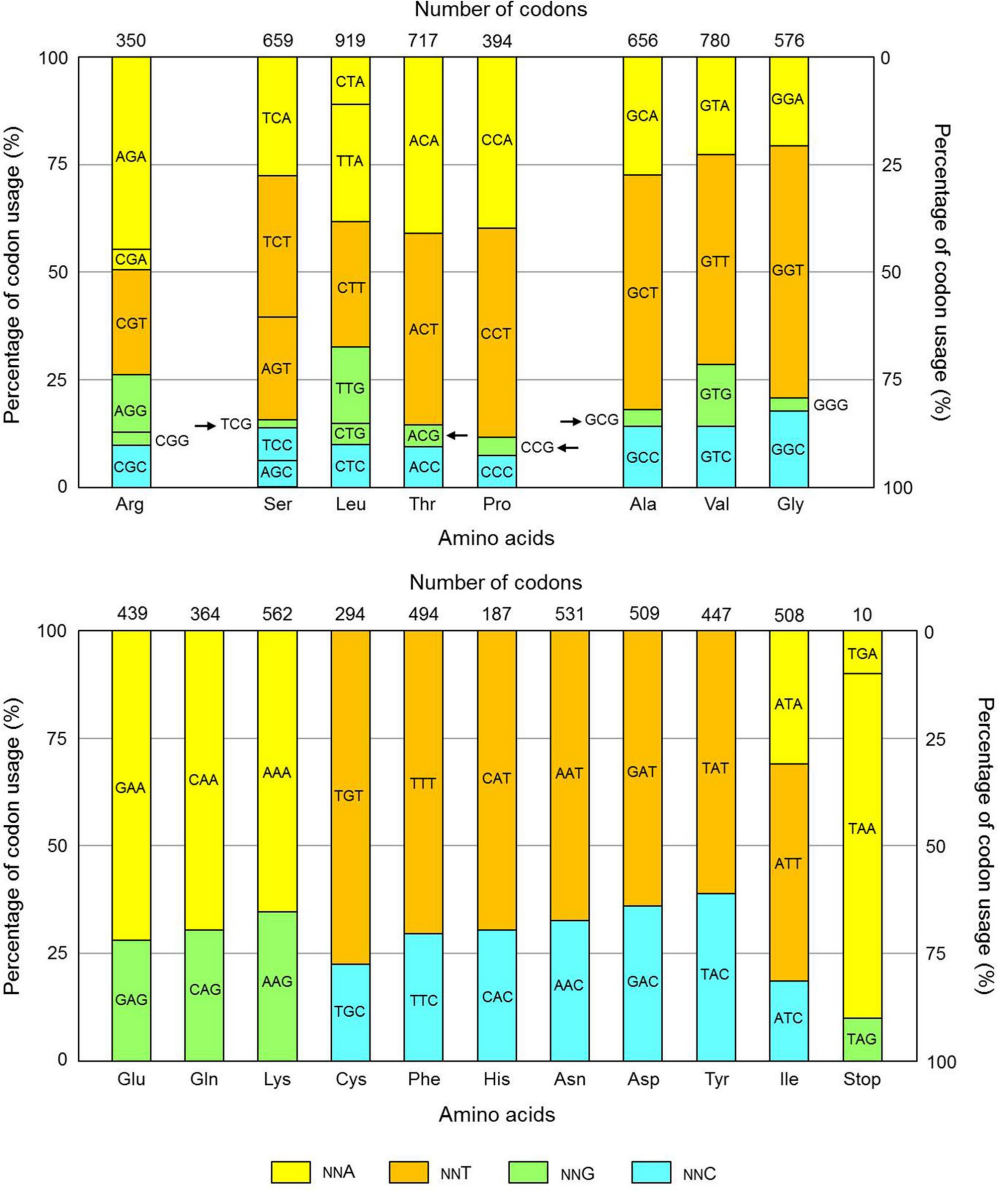
Percentages of codon usage in open reading frames of SARS-CoV-2. Usage of synonymous codons for eighteen amino acids (except methionine and tryptophan) and three stop codons are shown in the figure. Percentages of codons with A, T, G and C at codon position 3 are in yellow, brown, green and aqua blue background, respectively. Total number of codons for each amino acid is indicated at top of the percentage bar. Arrows indicate four codons that contain CG at positions 2 and 3.

The above-stated mutations are possible because very few of these mutations lead to changes in amino acids. To be specific, there are four codons containing (CG)_23_. They are TCG, CCG, ACG and GCG which code for serine, proline, threonine and alanine, respectively. Mutation of G at codon position 3 into T, C or A in all of them does not change the amino acid they encode. As for (CG)_31_, there are 16 codons having C at position 3. If this C is mutated into T, all 16 codons have the same meanings. And if it is mutated into A, 9 out of 16 codons still have the same meanings. Therefore, it is concluded that SARS-CoV-2 has evolved to reduce CG in ORFs mainly through mutating its G of (CG)_23_ and C of (CG)_31_ into A and T. Among them, C-to-T (i.e. C-to-U in RNA) occurs at a very high frequency probably because it is the simplest way to change a nucleotide (C becomes U after deamination). Besides, odds ratio of (CC)_23_ is much lower than that of (CA)_23_ and (CT)_23_ (Fig. 1c). This does not mean that (CG)_23_ has not been mutated into (CC)_23_. In fact, low odds ratio of (CC)_23_ is due to high frequency of C-to-T mutation at position 3, i.e. from (CG)_31_ into (TG)_31_ (Fig. 1d). The above views are also supported by codon usage bias in SARS-CoV-2 (Fig. 2), which shows that A/T-ended codons are much more frequently used than their synonymous G/C-ended codons. Besides, all four codons containing (CG)_23_ have the lowest percentages of usage among their correspondent synonymous codons.

### Low CG content in other coronaviruses

**Figure 3:**
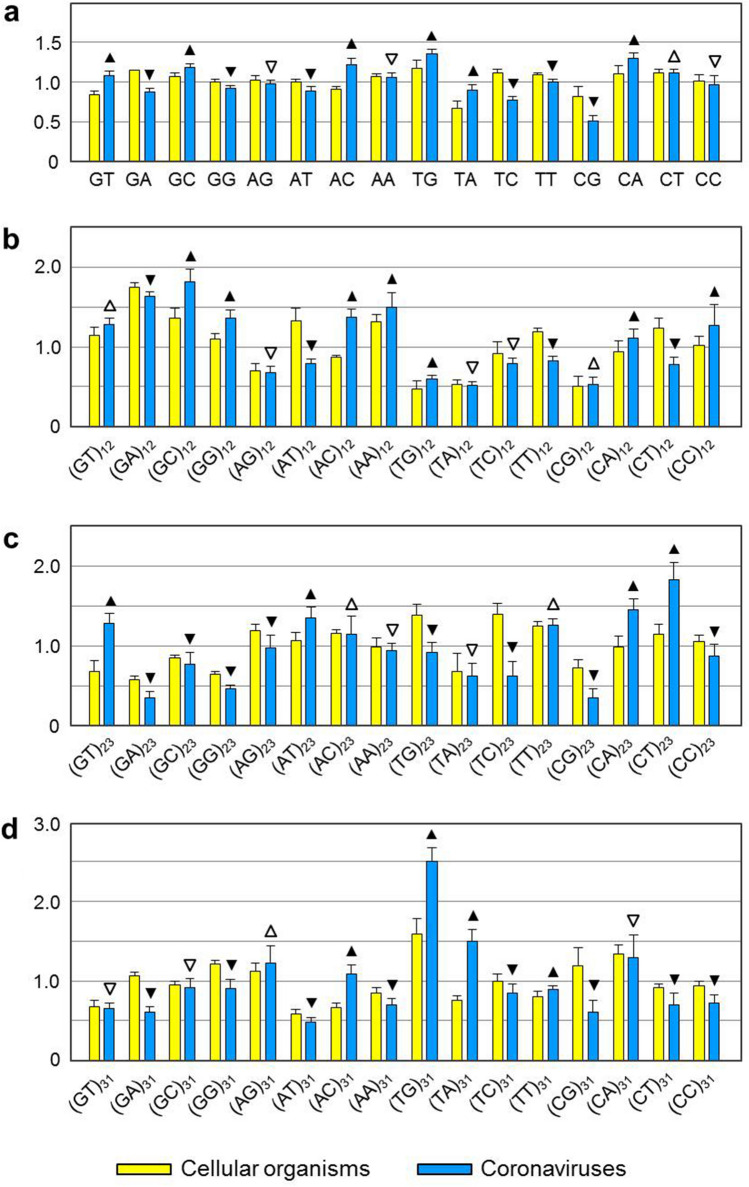
Odds ratios of dinucleotides in open reading frames of coronaviruses and cellular organisms. (**a**) odds ratios of dinucleotides at all codon positions. (**b**–**d**) odds ratios of dinucleotides at codon positions 1 and 2, 2 and 3, 3 and 1, respectively. Data of coronaviruses are from Table S1, which are shown in blue background. Those of cellular organisms are from our previous work^15^. Filled triangle or filled inverter triangle indicates that odds ratio of a dinucleotide in coronavirus is significantly higher or lower than that in cellular organisms at *p* = 0.05 level. Open triangle or open inverter triangle indicates that odds ratio of a dinucleotide in coronavirus is insignificantly higher or lower than that in cellular organisms.

**Figure 4:**
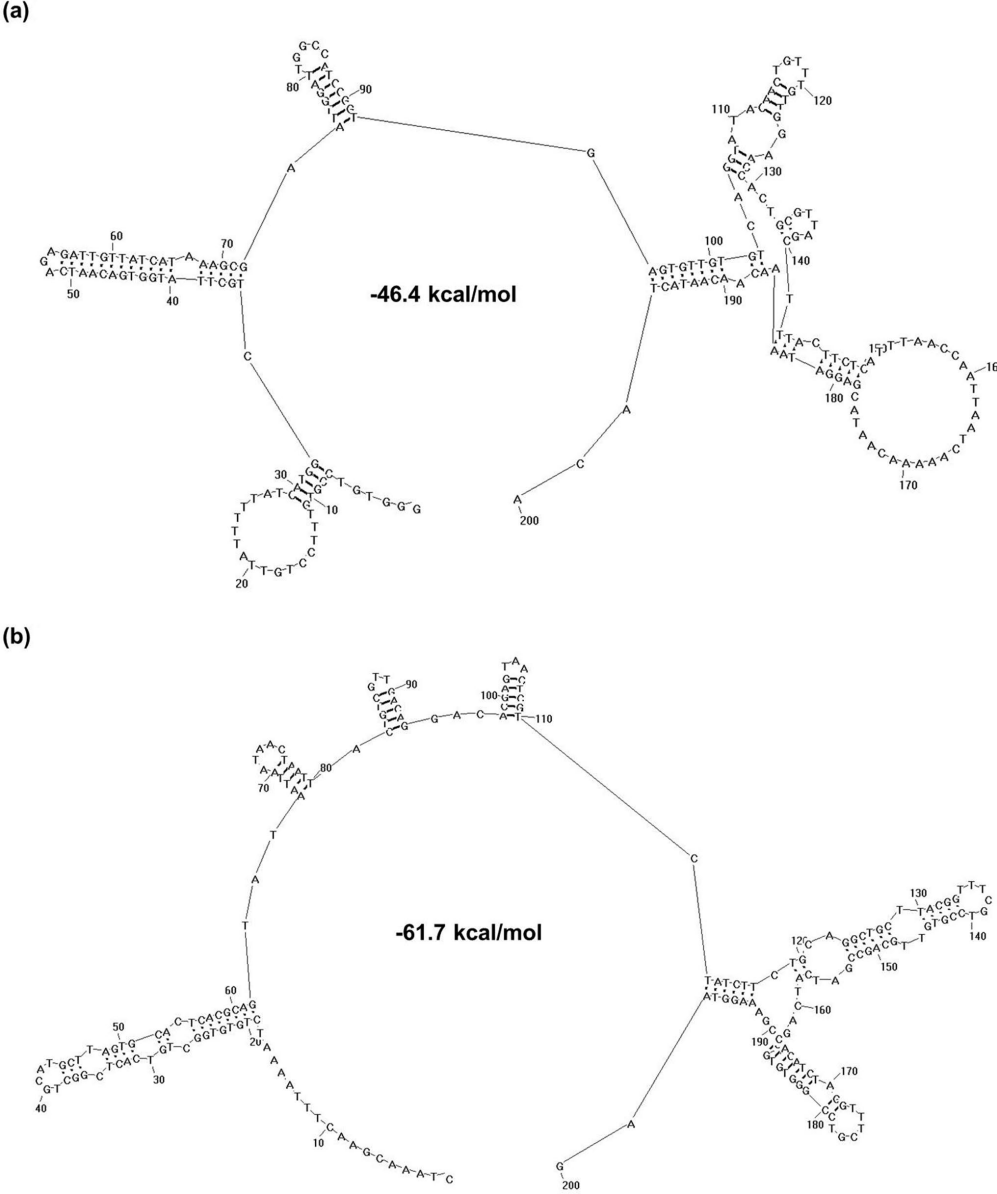
Secondary structure formed by 5′-UTR of poliovirus (**a**) and SARS-CoV-2 (**b**). The secondary structure is based on 200 nucleotides immediately upstream of the translation start site. Sequence number of poliovirus is MG212486. That of SARS-CoV-2 is NC_045512. Both structures and their free energy (indicated in centre of the structure) are drawn/calculated using RNAstructure (version 5.7)^27^.

Odds ratios of CG in ORFs of other coronaviruses are also very low (mean value = 0.50, Fig. 3 and Table S1). This could have profound effect on viral replication, because ORFs of coronaviruses are immediately translated by host ribosomes after being released into the cytoplasm of host cells^9^. The translation of viral RNA is affected by two factors. One is that host ribosomes must be recruited to the 5′-UTR (untranslated region) of viral RNA for initiation of translation. The other is that stem-loops formed by ORFs of viral RNA must be disrupted to expose coding information during translation. In contrast to ORFs, 5′-UTR of coronaviruses have quite high odds ratios of CG (mean value = 0.84, Table S2). This would facilitate formation of stable secondary structure that could serve as the internal ribosome entry site (IRES)^11–13^ for host ribosome (Fig. 4). Meanwhile, the viral RNA beginning at the translation start site (TSS) forms relatively unstable secondary structure, because its stem-loops are maintained by less hydrogen bonds (A-T and C-G base pairs have two and three hydrogen bonds respectively).

Stability variations of viral genomes at 5′-UTR and TSS-to-end regions could probably determine virulence of different viruses, because high stability of IRES structure means high efficiency in initiating translation, and high stability of TSS-to-end region means high energy consumption during translation. After high, medium and low stability of both 5′-UTR and TSS-to-end regions is given 3, 2 and 1 points respectively, virulence of coronaviruses can be classified into five grades, i.e. very high, high, medium, low and very low (Table 1). For example, human MERS (Middle East respiratory syndrome) coronavirus has very high virulence, because both its 5′-UTR and TSS-to-end regions are highly stable. High stability of 5′-UTR means that host ribosomes can be recruited to translate viral RNA at high rate. And, high stability of ORFs means that more energy is consumed to disrupt stem-loops in viral RNA during translation. Thus, normal translation of host cell mRNAs is greatly affected, suggesting that MERS coronavirus is highly virulent. SARS (severe acute respiratory syndrome) coronavirus has high virulence, because its 5′-UTRs is less stable than MERS coronavirus. SARS-CoV-2 has medium virulence, because it has medium stability in both 5′-UTR and TSS-to-end regions. This classification is consistent with estimations on case fatality ratio of MERS, SARS and COVID-19, which is 35%, 9% and 2.4% respectively^14^ and with our observations on odds ratio of CG in their ORFS, which is 0.56, 0.44 and 0.39 respectively (Table S1). Moreover, compared to SARS coronavirus, SARS-CoV-2 could infect and replicate more efficiently in human lung tissues but induce expression of less inflammatory cytokines/chemokines and mediators^15^. In our opinion, it is the lower C/G content in genomic RNA that allows SARS-CoV-2 to reproduce higher number of virus particles before triggering the immunoreaction of host cells, because less energy is consumed in replicating each virus particle.

**Table 1:** Stability of secondary structure formed by genome of coronavirus.

Genus	Virus	5′-UTR*		TSS-to-end		Virulence grade
		Free energy (kcal/mol)	Stability index	Free energy (kcal/mol)	Stability index	
Alphacoronavirus	Bat CoV CDPHE15	− 66.8	92 (H)	− 8,803.5	99 (H)	6
	Bat CoV HKU10	− 61.3	84 (M)	− 8,029.1	90 (H)	5
	Cat CoV1	− 68.8	95 (H)	− 7,963.0	89 (M)	5
	Rat CoV	− 59.5	82 (M)	− 8,615.0	97 (H)	5
	Mink CoV1	− 71.2	98 (H)	− 7,790.4	88 (M)	5
	Bat CoV1	− 59.6	82 (M)	− 8,153.4	92 (H)	5
	Bat CoV Sax2011	− 66.7	92 (H)	− 8,815.5	99 (H)	6
	Bat CoV SC2013	− 57.3	79 (L)	− 8,712.4	98 (H)	4
	PEDV	− 62.4	86 (M)	− 8,671.9	97 (H)	5
	Bat CoV HKU2	− 64.3	88 (M)	− 8,313.0	93 (H)	5
	Human CoV NL63	− 58.9	81 (M)	− 7,223.3	81 (M)	4
	Human CoV 229E	− 55.6	76 (L)	− 7,982.5	90 (H)	4
Betacoronavirus	Human CoV HKU1	− 43.1	59 (L)	− 6,864.6	77 (L)	2
	Human MERS-CoV	− 72.8	100 (H)	− 8,436.5	95 (H)	6
	Human SARS-CoV	− 63.2	87 (M)	− 8,054.1	91 (H)	5
	Human SARS-CoV-2	− 62.4	86 (M)	− 7,860.4	88 (M)	4
	Bat CoV ZJ2013	− 58.9	81 (M)	− 8,328.2	94 (H)	5
	Bat CoV HKU9	− 55.2	76 (L)	− 8,897.8	100 (H)	4
Deltacoronavirus	Wigeon CoV HKU20	− 51.8	71 (L)	− 8,273.2	93 (H)	4
	Bulbul CoV HKU11	− 54.3	75 (L)	− 8,387.6	94 (H)	4
	Heron CoV HKU19	− 54.9	75 (L)	− 7,687.2	86 (M)	3
	Moorhen CoV HKU21	− 51.2	70 (L)	− 8,140.4	91 (H)	4
Gammacoronavirus	Whale CoV SW1	− 62.8	86 (M)	− 8,161.3	92 (H)	5
	Turkey CoV	− 59.0	81 (M)	− 8,195.4	92 (H)	5

*Fee energy of 5′-UTR (untranslated region) was obtained by using 200 nucleotides immediately upstream of TSS (translation start site) for secondary structure prediction. Free energy of TSS-to-end region is normalized using the average genome size (28,085 nt) of all surveyed coronaviruses based on actual accumulated free energy of a specific genome (Table S2). 5′-UTR region of human MERS-CoV and TSS-to-end region of bat CoV HKU9 have the lowest free energy respectively, which are thus given the highest stability index (100). H (high), M (medium) and L (low) indicate stability index of ≥ 90, 80 to 89, and < 79, respectively. Virulence grade is based on stability of both 5′-UTR and TSS-to-end regions, in which H, M and L stability is given 3, 2 and 1 points respectively. For example, human SARS-CoV has M and H stability in 5′-UTR and TSS-to-end regions. Thus, its virulence is of grade 5 (i.e. 2 + 3). Various grades of virulence are interpreted as follows: 6—very high, 5—high, 4—medium, 3—low and 2—very low. MERS: Middle East respiratory syndrome. SARS: severe acute respiratory syndrome. PEDV: Porcine epidemic diarrhea virus. The viruses listed in the table were selected to represent different subgenera of coronaviruses.

Two other human coronaviruses have medium virulence as well. Among them, NL63 has medium stability in both 5′-UTR and TSS-to-end regions, whereas 229E has low stability in 5′-UTR but high stability in TSS-to-end region. Another human coronavirus (i.e. HKU1) has very low virulence, because it has low stability in both 5′-UTR and TSS-to-end regions (Table 1). The worldwide transmission of SARS-CoV-2 probably means that a coronavirus with medium virulence is more likely to spread rapidly. In comparison, a coronavirus with high or very high virulence could kill its host before causing severe epidemic, whereas a coronavirus with low or very low virulence is not able to replicate itself efficiently for further transmission.

## Discussion

**Figure 5:**
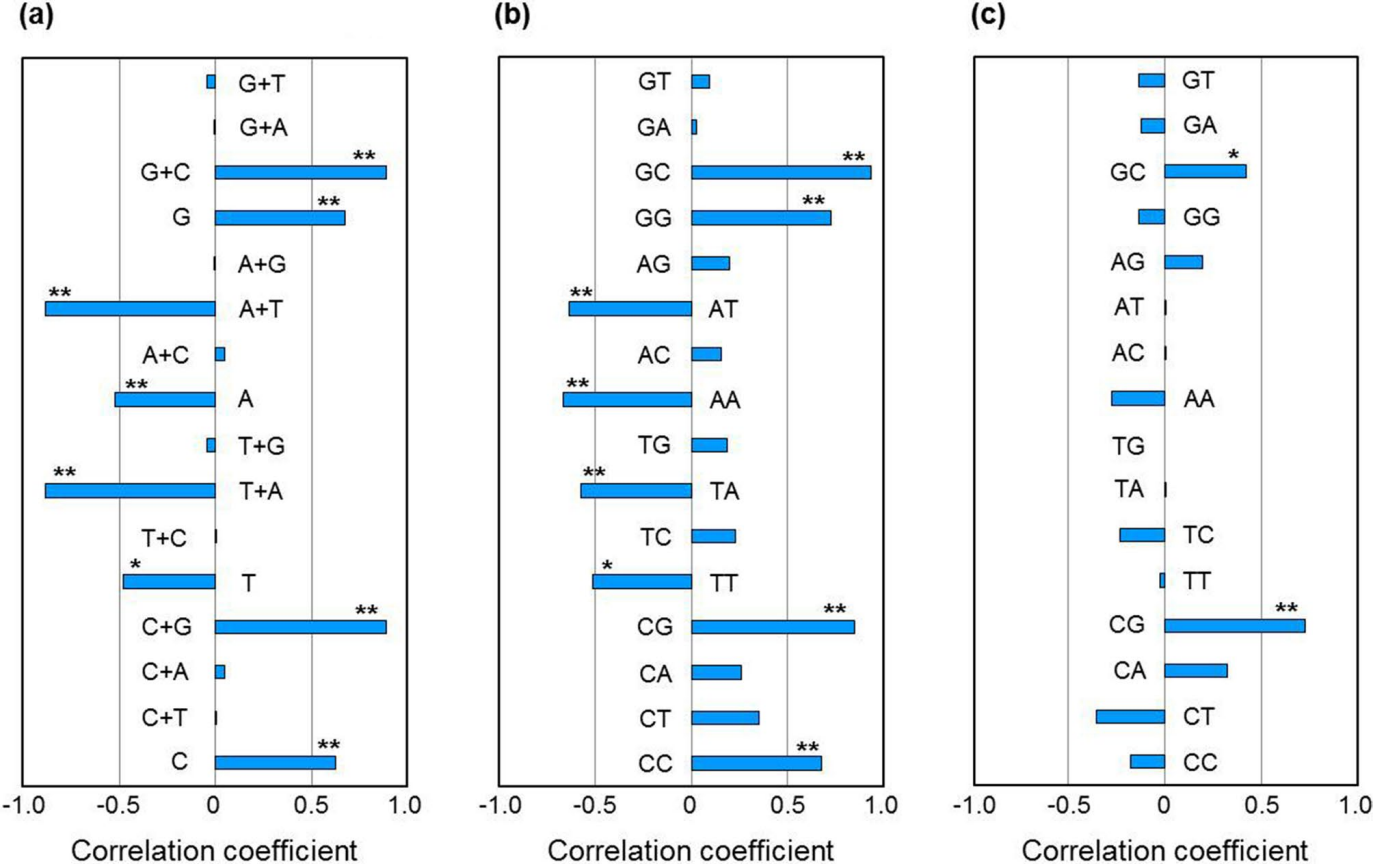
Correlation between RNA stability and nucleotide composition in viral genome. Shown here are correlation coefficients of RNA stability with (**a**) content of nucleotide(s), (**b**) content of dinucleotide and (**c**) odds ratio of dinucleotide in genomes of 24 coronaviruses. Only TSS-to-end region of viral genome is included for analysis (TSS: translation-start-site). * and **above data bar indicate that the correlation reaches significant (0.01 < *p* < 0.05) and extremely significant (*p* < 0.01) level, respectively. Detailed data for correlation analysis are listed in rows 67 to 103 of Table S2.

Our present study provides a novel insight into the evolution of human SARS-CoV-2. It is evident that this virus has evolved to reduce CG intensely in its ORFs. Such reduction is achieved mainly through mutating G of (CG)_23_ and C of (CG)_31_ into A or T (Fig. 1). Meanwhile, C or G not of CG may also be mutated. For example, TCA in SARS-CoV-2 of S-type has been mutated into TTA^16^. GTC and GGT in SARS-CoV-2 isolated from France have been mutated into TTC and GTT respectively^17^. Although the mutated C or G is not of CG and not at codon position 3, they do reduce C or G in viral RNA. C/G reduction is favourable for increasing efficiency of viral RNA translation, because stem-loops formed by less C/G-containing segments can be disrupted more easily. In fact, genomic RNA stability is closely related to nucleotide composition in coronaviruses (Fig. 5). First, RNA stability is positively correlated to content of C, G and C + G but negatively correlated to content of T, A and T + A (Fig. 5a). Second, RNA stability is also positively correlated to content of GC, GG, CG and CC but negatively correlated to content of AT, AA, TA and TT (Fig. 5b). Third, RNA stability is only positively correlated with odds ratio of dinucleotide GC and CG (Fig. 5c). As odds ratio measures the relative abundance of a specific dinucleotide, the extremely significant correlation between CG odds ratio and RNA stability strongly suggests that CG has been selected as the major target for mutation in coronaviruses.

Then, if reducing hydrogen bonds is the goal of base mutation, why is CG but not GC, GG or CC taken as the target for mutation? An examination on number of silent mutations of each dinucleotide at various codon positions reveals that CG has the highest number (47) of silent mutations among these four dinucleotides (Table 2 and Table S3). This explains why CG is the best target for mutation. Although CT has the same highest number like CG, it is not taken as the target for mutation because a T-to-C or T-to-G mutation would increase number of hydrogen bonds between potential base pairs, which is contradictory to the goal of mutation.

**Table 2:** Number of silent mutations of each dinucleotide at various codon positions.

Dinucleotide	Codon positions			Total
	1 and 2	2 and 3	3 and 1	
GT	0	8	30	38
GA	0	8	30	38
GC	0	8	30	38
GG	0	7	30	37
AG	4	4	32	40
AT	0	4	32	36
AC	0	4	32	36
AA	0	5	32	37
TG	1	7	33	41
TA	1	9	33	43
TC	2	9	33	44
TT	2	9	33	44
CG	2	12	33	47
CA	0	12	33	45
CT	2	12	33	47
CC	0	12	33	45

When a dinucleotide is located at codon positions 1 and 2 or at codon positions 2 and 3, there are four codons that contain this dinucleotide. Theoretically, they can be mutated into any of the rest 60 codons. When a dinucleotide is located at codon positions 3 and 1, only the nucleotide at position 3 is considered to mutate. There are 16 codons containing this nucleotide. Theoretically, they can be mutated into any of the rest 48 codons. Therefore, values in the table are number of silent mutations out of 60, 60 and 48 mutations for a dinucleotide at codon positions 1 and 2, 2 and 3, or 3 and 1, respectively.

It seems that the strategy of “reducing CG content to increase gene expression efficiency” has also been adopted by cellular organisms. As we have observed, CG in both ORFs and inter-genic regions of bacteria, archaea, fungi, plants and animals has an average odds ratio of 0.81, and that in introns of fungi, plants and animals is as low as 0.69. At time of our previous report^18^, we did not know why CG has such a low odds ratio in surveyed organisms. Now, after analysing cases in coronaviruses, we realize that low CG content in cellular organisms should also be the evolutionary consequence of increasing gene expression efficiency, because lowered CG content means reduced number of hydrogen bonds between DNA double strands (of the same length). Expression of a gene with low CG content saves energy not only in separating DNA double strands during transcription but also in disrupting stem-loops formed by mRNA during translation. Coincidently, CG is the very dinucleotide related to existence of CpG island, mutational hotspot, and single nucleotide polymorphism (SNP) in DNA sequences of cellular organisms. A CpG island is defined as a region of DNA with less methylated C, and this region generally contains actively expressed genes^19–21^. A mutational hotspot is defined as CG with methylated C, in which the methylated C is frequently mutated into T through deamination^22–24^. SNP refers to single nucleotide difference in genome sequences among individual organisms, which is observed most frequently at CG dinucleotide^25,26^. The relationship between CG reduction and these three important features of cellular DNA sequences is worthy of further investigations.

## Methods

Genome sequences of coronaviruses were retrieved from GenBank (www.ncbi.nlm.nih.gov). Odds ratios of dinucleotides were calculated using formulae developed by Karlin and Mrázek^10^ and by Wang et al.^18^ with self-compiled computer programs (C++ scripts are available upon request). Secondary structure and free energy of viral RNA is predicted using RNAstructure (version 5.7)^27^. SPSS software (version 17.0) was used to conduct independent-sample *t*-test for comparing difference in odds ratio of nucleotide between coronaviruses and cellular organisms, and to conduct correlation analysis between RNA stability and nucleotide composition in viral genomes.

## Supplementary information

Supplementary Table S1.

Supplementary Table S2.

Supplementary Table S3.
